# Non-Targeted Self-Measurement of Blood Pressure: Association with Self-Medication, Unscheduled Emergency Visits and Anxiety

**DOI:** 10.3390/medicina57010075

**Published:** 2021-01-17

**Authors:** Glessiane de Oliveira Almeida, Felipe J. Aidar, Dihogo Gama de Matos, Paulo Francisco de Almeida-Neto, Enaldo Vieira de Melo, José Augusto Soares Barreto Filho, Marcos Antonio Almeida-Santos, Victor Batista Oliveira, Rebeca Rocha de Almeida, Suelen Maiara dos Santos, Larissa Monteiro Costa Pereira, Juliana Santos Barbosa, Antônio Carlos Sobral Sousa

**Affiliations:** 1 Postgraduate Program in Health Sciences, Federal University of Sergipe, UFS, Sergipe 49100-000, Brazil; joseaugusto.se@gmail.com (J.A.S.B.F.); vbo.nutri@gmail.com (V.B.O.); rebeca_nut@hotmail.com (R.R.d.A.); contatosuelensantos@hotmail.com (S.M.d.S.); larissa_monteiroo@hotmail.com (L.M.C.P.); barbosa.juliana@live.com (J.S.B.); acssousa@terra.com.br (A.C.S.S.); 2 Group of Studies and Research of Performance, Sport, Health and Paralympic Sports (GEPEPS), Federal University of Sergipe, UFS, Sergipe 49100-000, Brazil; fjaidar@academico.ufs.br (F.J.A.); dihogogmc@hotmail.com (D.G.d.M.); 3 Department of Physical Education, Federal University of Sergipe, UFS, Sergipe 49100-000, Brazil; 4 Program of Physical Education, Federal University of Sergipe, UFS, Sergipe 49100-000, Brazil; 5 Program of Physiological Science, Federal University of Sergipe, UFS, Sergipe 49100-000, Brazil; 6 Health Sciences Center, Department of Physical Education, Federal University of Rio Grande do Norte, Natal UFRN 59064-741, Brazil; paulojitte@ufrn.edu.br; 7 Department of Medicine, UFS), Federal University of Sergipe, UFS, Sergipe 49100-000, Brazil; evmsidarta@gmail.com (E.V.d.M); marcosalmeida2010@yahoo.com.br (M.A.A.-S.); 8 Teaching and Research Center of São Lucas Hospital/Rede D’Or—São Luiz de Aracaju, Sergipe 49015-400, Brazil; 9 Division of Cardiology, University Hospital of Federal University of Sergipe, UFS, Sergipe 49100-000, Brazil; 10 Postgraduate Program in Health and Environment, Tiradentes University, UNIT, Aracaju, Sergipe 49032-490, Brazil

**Keywords:** systemic arterial hypertension, self-measurement, self-medication, anxiety

## Abstract

*Background and Objective:* The routine practice of self-medication of blood pressure (BP) not oriented with pulse devices may not be precisely useful in the control of BP and can lead the patient to self-medicate in error. Thus, we need to evaluate the non-oriented self-assessment of BP in real-life circumstances in hypertensive patients. The objective of this study was to evaluate in hypertensive patients the association of BP self-measurement with its control, as well as the presence of anxiety disorders, the occurrence of unscheduled visits to the emergency room, and self-medication. *Materials and Methods:* An observational study was carried out with 1000 hypertensive volunteers (age: 61.0 ± 12.5). Using a questionnaire, sociodemographic and clinical data on BP control were collected. Anxiety was assessed by the State-Trait Anxiety Inventory (STAI). *Results:* The group that performed non-oriented self-measurement of BP, showed that they had higher frequencies of self-medication (57.9%, *p* < 0.05) and more unscheduled visits to the emergency room (68%, *p* < 0.05). In addition, a lower level of BP control (46.8%, *p* < 0.05) was associated with higher levels of anxiety (52.3%, *p* < 0.05) in the group that performed non-oriented self-measurements of BP. *Conclusion:* The practice of non-oriented self-assessment of BP was associated with negative factors such as high levels of anxiety and higher frequencies of self-medication and unscheduled emergency visits.

## 1. Introduction

Systemic arterial hypertension is considered a public health problem because it is associated with a high risk of mortality [1]. Factors such as self-medication [2], self-measurement and BP control influence non-adherence to treatment due to a lack of knowledge and guidance [3,4]. There are several pharmacological and non-pharmacological therapeutic measures that can be applied to control hypertension. However, the general population’s knowledge of hypertension and BP control is still not ideal and, therefore, effective strategies must be developed to improve BP control and treatment adherence [4].

One of the strategies used by hypertensive patients to achieve these goals is self-measurement of BP at home with the aid of digital devices. Some studies have shown that BP self-measurement allows for a better and/or similar BP prognosis and control than measurements performed in a health care setting [5,6,7,8]. In addition, BP self-measurement is more attractive to the patients than the usual procedure of making medical appointments [4]. However, despite the patient’s preference for using the digital device to measure systemic blood pressure, whether for the convenience of carrying out a greater number of measurements during the day, the reliability of BP measurements, that is, the measurement performed by the patient himself, is not unanimously accepted [4,9]. This is due to the great variation that occurs in BP due to the lack of posture during the measurement, interferences of the situations experienced by the patient, exposure to stressful events, such as anxiety, throughout the day [10].

Studies indicate that this self-measurement procedure, when controlled and instructed by the clinician, has positive effects on the diagnosis and adherence to the patient’s treatment [11,12,13], other studies [4,9] indicate that it interferes with the control of BP. Such researches aimed to evaluate self-measurement of blood pressure in a controlled and instructed way, regarding the position and indication of correct measurements, by health professionals. However, these investigations were not conducted with the aim of evaluating the association of BP self-measurement in patients with anxiety disorder, for example. It is known that anxiety is one of the factors that influence the increase in blood pressure. Thus, it is suggested that hypertensive patients with anxiety disorder and who perform self-measurement may result in higher occurrences of unscheduled visits to the emergency room and self-medication.

Therefore, the present study aimed to evaluate in hypertensive patients the association of BP self-measurement with its control, as well as the presence of anxiety disorders, the occurrence of unscheduled visits to the emergency room, and self-medication.

## 2. Methods

**Figure 1:**
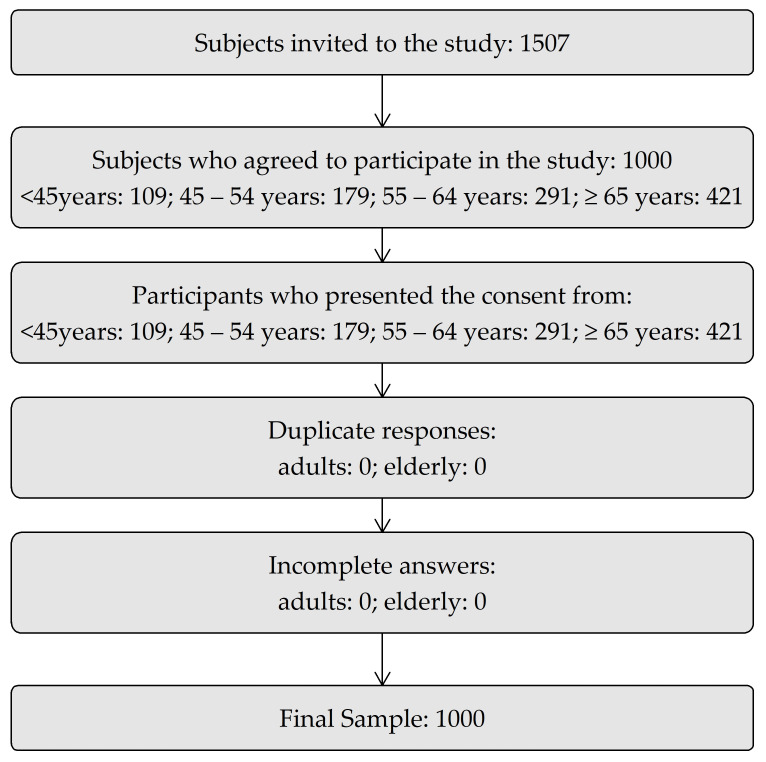
Sample recruitment.

The present study was characterized as cross-sectional and observational with an analytical character, carried out between June 2017 and October 2019 in the city of Aracaju-Sergipe, Brazil. The sample was carried out in a non-random manner with the evaluation of 1000 consecutively selected patients to minimize sampling bias. As an inclusion criterion, patients from 18 years of age, both sexes and diagnosed with systemic arterial hypertension were defined. Those with mental disorders that could compromise the answers to the questionnaires were excluded. Initially, 1507 subjects were invited to participate in the research and 1000 responded that they would accept it. It was found that there were no duplicate or incomplete responses, and 1000 responses were readable for the final analysis (Figure 1). The classification of age groups was defined based on intervals used by Wang et al. [14]. The sample was divided into the following groups: <45 years, (39.57 ± 4.28; 57% female and 43% male), 45–54 years (49.71 ± 2.89; 55% female and 45% male), 55–64 years (59.80 ± 2.98; 56% female and 44% male) and ≥65 years (72.49 ± 6.06; 59% female and 41% male).

Data collection was performed through the application of a specific questionnaire for research, which included data related to the patient’s sociodemographic and clinical aspects, quantification of unscheduled visits to the emergency services, self-medication and BP self-assessment. Self-medication related to antihypertensive treatment was considered as the use by the patient of an extra dose of an antihypertensive without a recommendation by a health care professional, the use of another non-prescribed antihypertensive, the non-use of an antihypertensive or not following the prescribed dose. The criterion for a self-measurement was the patient’s report of their frequency of use of the blood pressure (BP) measuring device per day and/or week. An unscheduled visit to the emergency room was based on the patient’s report of visits to the emergency room because of high BP in the last 12 months, as confirmed by their medical records. The control of BP was defined by means of ambulatory blood pressure monitoring (ABPM) or the average of the measurements in the last three consultations that were taken by three assistant physicians according to the College of Cardiology and the cardiology guidelines.

The State-Trait Anxiety Inventory (STAI) was applied by a trained psychologist and information on clinical data and patient identification was collected by a nurse at the Federal University of Sergipe.

The sample consisted of hypertensive patients aged 18 or over, of both sexes, followed up on an outpatient basis at three hospital institutions in the city of Aracaju-Sergipe, Brazil. One of these institutions exclusively serves users of the public health service and two serve the private sector. Those diagnosed with a mental disorder based on the answers to the questionnaires were excluded from the research. Patients who consented to participate in the present study signed an Informed Consent Form. This study was approved by the Research Ethics Committee involving human beings under the number CAAE: 60473316.9.0000.5546.

### 2.1. Procedures

Data collection was performed through the application of a specific questionnaire covering the following topics: (1) Patient identification and sociodemographic elements (gender, age, income, education, marital status, self-medication, unscheduled visits to the emergency room, information on BP self-checking). The social class and education were inserted in the questionnaire according to the classification used by the Brazilian Institute of Geography and Statistics (IBGE). IBGE is a public institute of the Brazilian federal administration, which provides the geographic and statistical information of Brazil and classifies the social class by family income group, in which class A corresponds to above 20 minimum wages; B: 10 to 20 minimum wages; C: 4 to 10 minimum wages; D: 2 to 4 minimum wages; E: Up to 2 minimum wages. Education is classified as: elementary, high school, university, graduation program and never studied. (2) Clinical data: a cardiologist evaluation based on the average of the last three measurements performed in the last three consultations according to the College of Cardiology and the cardiology and/or ABPM guidelines for the classification of controlled and uncontrolled BP, in addition to the identification of the comorbidities of such patients. BP values were considered for the diagnosis of systemic arterial hypertension (SAH) according to the recommendations of the 2017 guideline, in which the American Society of Cardiology [15] classifies blood pressure levels differently and suggests a definition for stage 1 blood pressure values. Systolic blood pressure (SBP) between 130–139 mmHg or diastolic blood pressure (DBP) between 80–89 mmHg; Stage 2 hypertension includes subjects with SBP values greater than 140 mmHg or DBP equal to or greater than 90 mmHg. The category of normal blood pressure was defined as SBP less than 120 mmHg and DBP less than 80 mmHg, and elevated BP was classified as SBP between 130–139 mmHg and DBP higher than 90 mmHg. This categorization is justified based on observational data related to the association between diastolic and systolic blood pressure and risk of cardiovascular diseases. To dichotomize the variable in controlled and uncontrolled hypertension, the procedure was carried out through ABPM or by the average of the measurements from the last three consultations performed by three medical assistants according to the College of Cardiology and the guidelines of Brazilian cardiology [16]. The cutoff point for uncontrolled hypertension was between SBP: 130–139 mmHg or DBP between 80–89 mmHg. Anxiety: the STAI was applied by a trained psychologist to all research volunteers. STAI aims to assess anxiety as a characteristic of the state (E) and personality (T). It is a self-assessment instrument, comprising two parallel scales, each with 20 items [17]. On STAI, on the T scale according to the sieve, the stipulated average is 45.34 to 55.22 and on the E scale the expected average is 43.64. The internal consistency of both scales was determined based on Cronbach’s alpha. The E scale showed a Cronbach’s alpha of 0.91, while the T scale was 0.894. Therefore, the instruments showed high internal validity when compared to the general population, with an index between 5.6% and 1.8%. Values above 0.8 indicate a high consistency, although coefficients above 0.60 have demonstrated adequate consistency [18].

### 2.2. Statistics

The continuous variables were described as mean and standard deviation. As for the categorical variables, absolute frequencies and percentages, and 95% confidence intervals were used to summarize them when relevant. The Shapiro–Wilk test was used to assess the assumption of normality. To test hypotheses related to categorical variables, Pearson’s chi-square test or Fisher’s exact test were used when most appropriate. The comparison between groups (with self-assessment versus without self-assessment; controlled vs. uncontrolled hypertension) was performed using Student’s *t* test for independent data in the case of quantitative variables. The Breslow–Day test was applied to assess whether the intensity of the relationship between anxiety (trait and state) and gender is dependent on the age groups. To analyze the factors associated with the outcome variables (self-measurement of systemic BP, uncontrolled hypertension, self-medication, unscheduled visits and trait anxiety), the logistic regression technique was used using the “forward stepwise” and “backward stepwise” method, considering entry in the model *p* = 0.25 and remaining in the model *p* = 0.05. Then, simple and adjusted odds ratios were calculated. The Statistical Package for Social Sciences version 24.0 was used to perform the statistical calculations for testing. The estimates were made with the following parameters: power = 80%. The effect size (Cohen’s D for continuous variables and Cohen’s h for categorical variables) was defined as small (<0.20), medium (between 0.20 and 0.50), large (between 0.50 and 0.80) and very large (>1, 20) [19]. The two-tailed p value less than 0.05 was taken as the criterion of statistical significance.

## 3. Results

The patients had a mean age of 61.0 ± 12.5 with a minimum of 27 years and a maximum of 100 years. It was observed that 50% of the patients were followed up in the cardiology outpatient clinic of the Teaching Hospital of the Federal University of Sergipe and the other half in the supplementary network. The clinical characteristics shown in Table 1 are typical of this patient population.

### 3.1. Variables Outcomes

It was observed during the study period that there was a high frequency of self-measurement of BP (44.7%), uncontrolled hypertension (36.8%), self-medication (41.3%), unplanned visits to the emergency room (38.4%), and anxiety (51.6%), as shown in Table 2.

### 3.2. Difference between Sociodemographic and Clinical Characteristics of Patients who Self-Measured Blood Pressure (BP)

Table 3 shows the data of patients with and without self-measurement of blood pressure in relation to sociodemographic characteristics. Self-measurement significantly associated with social class was observed.

The data of patients with and without self-measurement of blood pressure in relation to sociodemographic characteristics by age groups can be seen in Table A1. It was observed self-measurement significantly associated with gender (45–54 years), marital status (≥65 years) and social class (<45 years).

### 3.3. Comparison between Self-Assessment and Non-Self-Assessment

Data from patients with and without self-measurement of blood pressure in relation to clinical characteristics is shown in Table 4. Self-measurement significantly associated with comorbidities, diabetes mellitus, dyslipidemia, coronary artery disease, stroke, peripheral obstructive arterial disease, use of medication for comorbidities, self-medication, unscheduled visit, BP control, and anxiety state was observed.

Data from patients with and without self-measurement of blood pressure in relation to clinical characteristics age groups was showed in Table A2. It was observed that self-measurement was significantly associated with self-medication, unscheduled visit and BP control for patients under 45 years old, dyslipidemia, self-medication, unscheduled visit, BP control and anxiety state for 45- to 54-year-old patients, comorbidities, diabetes mellitus, use of medication for comorbidities, self-medication, unscheduled visit, BP control and anxiety state for 55- to 64-year-old patients and diabetes mellitus, dyslipidemia, coronary artery disease, peripheral obstructive arterial disease, self-medication, unscheduled visit and BP control for patients above 65 years old.

### 3.4. Difference between Sociodemographic and Clinical Characteristics of Patients with Controlled and Uncontrolled BP

The values of patients with controlled BP and uncontrolled BP in terms of sociodemographic characteristics, are shown in Table 5. Controlled BP significantly associated with gender was observed.

The values of patients with controlled BP and uncontrolled BP in terms of sociodemographic characteristics by age groups, as shown in Table A3. It was observed that controlled BP was significantly associated with education in <45-year-old patients and gender in 55- to 64-year-old patients and ≥65-year-old patients.

The values of patients with controlled and uncontrolled BP in terms of clinical characteristics are seen in Table 6. Controlled BP significantly associated with type of hospital, comorbidities, diabetes mellitus, dyslipidemia, peripheral obstructive arterial disease, use of medication for comorbidities, self-medication, unscheduled visit, and trait and anxiety state was observed.

The values of patients with controlled and uncontrolled BP in terms of clinical characteristics by age group, are seen in Table A4. It was observed that BP-control was significantly associated with stroke, unscheduled visit and anxiety traits in <45-year-old patients, dyslipidemia, use of medication for comorbidities, unscheduled visit and trait anxiety state for 45- to 54-year-old patients, comorbidities, diabetes mellitus, dyslipidemia, use of medication for comorbidities, and trait anxiety state for 55- to 64-year-old patients, comorbidities, diabetes mellitus, dyslipidemia, peripheral obstructive arterial disease, Use of medication for comorbidities, self-medication and unscheduled visits for ≥65-year-old patients.

In Table A5, the association between anxiety (trait and state), gender and age groups was observed. It was seen an association between gender and trait anxiety in <45-, 55–64, ≥65-year-old and in general and with state anxiety in <45-, ≥65-year-old and in general. There was an effort to identify whether the association of trait and state anxiety and gender are dependent of age groups applying the Breslow-Day test. The p-values of 0.423 for state anxiety and 0.187 for trait anxiety were observed, which lead us to believe that the relationship between gender and anxiety are independent of age group. The conclusion was that female patients are more anxious (trait and state) than Male patients independent of age groups.

### 3.5. Association between Anxiety and Gender by Age Groups

In multivariable logistic regression, the factors associated with non-BP control were: self-measurement, self-medication, unscheduled visits, state anxiety, a female prevalence, the presence of comorbidities, the use of medication for comorbidities and trait/state anxiety (Table 7).

The odds ratio of not BP and trait anxiety and state anxiety controlled for gender, use of medication for comorbidities, self-measurement of BP, self-medication and state anxiety or trait anxiety respectively were estimated (Table 8). It was noted that state/trait anxiety was an associated factor of not controlling arterial hypertension even on the presence of gender, use of medication for comorbidities, self-measurement of BP, and self-medication.

## 4. Discussion

BP measurement is an important procedure that must be performed for any medical evaluation, regardless of specialty [20], and in view of the previous information, the main findings of this study were that patients who performed self-measurements had less control over their BP, self-medicated more frequently, had a greater presence of state anxiety and attended the emergency room more frequently because of their BP. These findings seem to point out that there is a lack of knowledge on the part of patients, regarding the self-measurement of blood pressure, and its implications when performing this procedure. Thus, a more effective explanation of how to use the pressure device would minimize the worsening of existing diseases.

The result of this research differs from some studies [4,21,22,23] on self-measurement and BP control. In the aforementioned studies, patients were instructed on the use, calibration and validation of the blood pressure device before use, in addition to the correct position and BP values for control. However, in the present study, the sample was observed in real-life circumstances, with no guidance on how to use the device. In this case, the objective was to observe how the population has been using the blood pressure device without guidance. Another important point is that factors such as association with anxiety, self-medication and visits to the emergency room were not investigated in the studies cited.

Research has shown that women constitute the majority of the hypertensive population followed in primary health care [9,24]. Thus, we found that women have higher frequencies of BP self-measurement (60.2%), that is, they seek greater health care. On the other hand, they presented worse BP control (65.2%). Data from the National Survey on Access, Use and Promotion of Rational Use of Medicines in Brazil (PNAUM) point out that females have a greater influence on the practice of self-medication [25], a predictive factor for non-adherence to medication [2] and, consequently, a factor that implies the lack of BP control. In addition, the appearance of physical and psychological disorders such as anxiety, insomnia, tiredness, and irritability are more common in women than in men, in addition to the decrease in the production of estrogens, changes in the lipid profile, weight gain and sedentary lifestyle [9].

One of the main causes of self-medication is the need to relieve symptoms [26] and psychosocial factors [27]. Among the most described symptoms are headache, atypical chest pain, dyspnea, acute psychological stress, anxiety and panic syndrome. When patients associate these symptoms with high BP, this condition is characterized as a false hypertensive crisis [1]. Among psychiatric disorders, anxiety is the most prevalent in the general population, with prevalence rates between 5.6% and 18.1% [28]. In women, this prevalence is higher than in men, being a risk factor for elevated BP [9,29,30,31], which can constitute a barrier to non-BP control [9,32]. According to a study by the Global Burden of Disease [33], the sixth leading cause of disability in the world is related to mental disorders, and individuals affected with anxiety symptoms have lower quality of life and worse psychosocial factor. Individuals with such symptoms tend to present a pattern of recurrence to the disorder and an increasing urgency leading to a chronic course and worsening of other illnesses.

In some studies that analyzed the profile of individuals who seek health services in this country, it was observed that there is a predominance of users who seek urgent care with chronic diseases, such as arterial hypertension, with greater severity due to their lack of control of the condition [34,35].

It needs to be taken into account that hypertension is the main treatable cardiovascular risk factor [36,37,38]. Hypertension tends to significantly increase the risk of myocardial infarct, stroke, kidney damage, and other pathologies [39]. In this sense, ineffective control, especially of hypertension, and cardiovascular problems, would be linked to therapeutic inertia, the use of incorrect dosages and/or inappropriate combinations of medication, low adherence to treatment [3], an unhealthy lifestyle (smoking, alcohol abuse, excess of fat and salt in the diet, sedentary habits, and being overweight), use of self-assessments in medical guidance and indication, and the prescription of other drugs that can induce hypertension, even when self-administered.

## 5. Study Limitation

Regarding the limitations of this study, it should be noted that there was no randomization for the use or non-use of the blood pressure device because the devices used were obtained by the patients, which can lead to errors resulting from the use of the device, mistakes regarding knowledge about the device and other distortions.

Another limitation of the study is that there was no standardization regarding the performance of ABPM, since not all patients underwent the exam which can interfere with some results. It is suggested for future work, randomized studies to assess the influence of self-measurement with self-medication, unscheduled visits to the hospital and anxiety disorders.

## 6. Conclusions

Patients who self-assessed BP had the lowest blood pressure control, self-medicated more frequently, had a greater presence of state anxiety and had more emergency room visits because of their blood pressure. The factors associated with non-BP control were: self-medication, unscheduled visits to the hospital, state of anxiety, prevalence of females, the presence of comorbidities, especially diabetes mellitus and dyslipidemia, and the use of medications for comorbidities.

Thus, the lack of knowledge about the disease and its implications, as well as the wrong guidance on the use of the pressure device are key factors in this chain of events and not BP control. Therefore, it is wise to discourage the use of digital pressure devices in patients to whom adequate guidance has not been given and the clinical picture has been observed, especially if such a patient suffers from an anxiety disorder. In addition, it is wise to invest in public policies aimed at capacitors, informing the population about the proper use of the blood pressure device and pointing out the importance of a multidisciplinary approach in the management of hypertensive patients with anxiety disorder.

## Figures and Tables

**Table 1:** General characteristics of hypertensive patients.

Characteristics	Values
**Age**	
<45	109 (10.9%)
45–54	179 (17.9%)
55–64	291 (29.1%)
≥65	421 (42.1%)
**Social class**	
A	78 (7.8%)
B	131 (13.1%)
C	180 (18%)
D	243 (24.3%)
E	368 (36.8%)
**Marital Status**	
Married	607 (60.7%)
Divorced	133 (13.3%)
Single	139 (13.9%)
Widowed	91 (9.1%)
Live with a partner	30 (3.0%)
**Education**	
Never studied	82 (8.2%)
Fundamental	291 (29.1%)
High school	315 (31.5%)
University	271 (27.1%)
Graduate Studies	41 (4.1%)
**Comorbidities**	
Yes	523 (52.3%)
No	368 (36.8%)
**Use of medicines for comorbidities**	
Yes	464 (46.4%)
No	535 (53.5%)

Values expressed in absolute frequencies (n) and percentage in parentheses (%). A = High social class, B = High middle class. C = Middle social class. D = low middle class. D = Poor social class.

**Table 2:** Estimation of frequency of self-measurement, control of arterial hypertension, self-medication and Unscheduled urgent visit.

	Values	95 % CI
**HOSPITAL**		
Public	500 (50.0 %)	-----
Private	500 (50.0%)	-----
SBP Public (mm Hg)	137.6 ± 15.6	136.2–138.9
DBP Public (mm Hg)	78.5 ± 13.3	77.3–79.6
SBP Private (mm Hg)	133.1 ± 13.4	132.0–134.3
DBP Private (mm Hg)	77.6 ± 13.0	76.4–78.7
Self-measurement	447 (44.7%)	41.8–47.7
Controlled hypertension	632 (63.2%)	60.0–66.1
Self-medication	413 (41.3%)	38.5–44.3
Unscheduled urgent visit	384 (38.4%)	35.3–41.5
**Anxiety**		
Trait	516 (51.6%)	48.6–54.7
State	457 (45.7%)	42.8–48.9

Values expressed in n (%) and 95% CI = 95% confidence interval. SBP = systolic blood pressure. DBP = diastolic blood pressure.

**Table 3:** Comparison between patients with and without self-measurement of blood pressure regarding sociodemographic characteristics.

	Self-Measurement			
Sociodemographic	With	Without	*p*	h
Characteristics	(n = 447)	(n = 553)		
**Age (years)**	61.1 (12.2)	61.0 (12.7)	0.98	0.008
<45	47 (10.5)	62 (11.2)	0.872	−0.022
45–54	76 (17)	103 (18.6)		−0.042
55–64	134 (30)	157 (28.4)		0.035
≥65	190 (42.5)	231 (41.8)		0.015
**Hospital**				
Public	219 (49.0)	281 (50.8)	0.567	−0.036
Private	228 (51.0)	272 (49.2)		0.036
**Gender**				
Female	269 (60.2)	302 (54.6)	0.077	0.113
Male	178 (39.8)	251 (45.4)		−0.113
**Marital Status**				
Married	271 (60.6)	336 (60.8)	0.904	−0.003
Divorced	62 (13.9)	71 (12.8)		−0.002
Single	58 (13.0)	81 (14.6)		−0.048
Widowed	41 (9.2)	50 (9.0)		0.004
Live with a partner	15 (3.4)	15 (2.7)		0.037
**Social class**				
A	29 (6.5)	49 (8.6)	0.017	−0.089
B	47 (10.5)	84 (15.2)		−0.14
C	93 (20.8)	87 (15.7)		0.131
D	120 (26.8)	123 (22.2)		0.107
E	158 (35.3)	210 (38.0)		−0.054
**Education**				
Never studied	37 (8.3)	45 (8.1)	0.592	0.005
Fundamental	129 (28.9)	162 (29. 3)		−0.01
High school	143 (32.0)	172 (31.1)		0.019
University	115 (25.7)	156 (28.2)		−0.056
Graduate Studies	23 (5.1)	18 (3.3)		0.095

Age expressed as mean and standard deviation; other data expressed in absolute numbers and percentage in parentheses; *p*: statistical significance (chi-square test and Student’s *t*-test). A = high social class, B = high middle class. C = middle social class. D = low middle class. D = poor social class. h—Effect size Cohen’s D for continuous variables and Cohen’s h for categorical variables.

**Table 4:** Comparison between patients with and without self-measurement of blood pressure regarding clinical characteristics.

	Self-Measurement			
Clinical Features	With	Without	*p*	h
	(n = 447)	(n = 553)		
Comorbidities	256 (57.3)	267 (48.3)	0.005	0.18
Diabetes mellitus	120 (26.8)	96 (17.4)	<0.001	0.23
Dyslipidemia	182 (54.5)	158 (28.6)	<0.001	0.256
Coronary artery disease	63 (14.1)	53 (9.6)	0.026	0.14
Chronic kidney disease	7 (1.6)	13 (2.4)	0.378	−0.057
Stroke	29 (6.5)	17 (3.1)	0.01	0.162
Depression	11 (2.5)	11 (2.0)	0.613	0.032
Cardiac insufficiency	17 (3.8)	28 (5.1)	0.339	−0.061
Chronic obstructive pulmonary disease	9 (2.0)	7 (1.3)	0.349	0.059
Peripheral obstructive arterial disease	19 (4.3)	6 (1.1)	0.001	0.207
Use of medication for comorbidities	223 (49.9)	241 (43.7)	0.05	0.126
**Purchase of the medicine**				
Health center (free)	144 (32.2)	179 (32.4)	0.155	−0.029
Popular pharmacy	112 (25.1)	112 (20.3)		0.096
Pharmacy (full amount)	191 (42.7)	262 (47.4)		−0.129
Self-medication	259 (57.9)	154 (27.8)	<0.001	0.115
Unscheduled visit	304 (68)	80 (14.5)	<0.001	−0.093
BP control	209 (46.8)	423 (76.5)	<0.001	0.618
**Anxiety**				
Trait	241 (53.9)	275 (49.7)	0.188	0.084
State	234 (52.3)	223 (40.3)	<0.001	0.242

Data expressed in absolute numbers and percentage in parentheses; *p*: statistical significance (chi-square test). h—Effect size Cohen’s h.

**Table 5:** Comparison between patients with controlled BP and uncontrolled BP in terms of sociodemographic characteristics.

	Controlled BP			
Characteristics	Yes	No	*p*	h
	(n = 632)	(n = 368)		
**Age (years)**	60.5 ± 12.6	62.0 ± 12.2	0.058	0.121
<45	76 (12)	33 (9)	0.174	0.1
45–54	121 (19.1)	58 (15.8)		0.089
55–64	180 (28.5)	111 (30.2)		−0.037
≥65	255 (40.3)	166 (45.1)		−0.096
**Gender**				
Female	331 (52.4)	240 (65.2)	<0.001	−0.262
Male	301 (47.6)	128 (34.8)		0.262
**Marital Status**				
Married	386 (61.1)	221 (60.1)	0.82	0.021
Divorced	83 (13.1)	50 (13.6)		−0.001
Single	90 (14.2)	49 (13.3)		0.027
Widowed	57 (9.0)	34 (9.2)		−0.008
Live with a partner	16 (2.5)	14 (3.8)		−0.073
**Social class**				
A	51 (8.1)	27 (7.3)	0.33	0.027
B	93 (14.7	38 (10.3)		0.133
C	113 (17.9)	67 (18.2)		−0.008
D	147 (23.3)	96 (26.1)		−0.066
E	228 (36.1)	140 (38.0)		−0.041
**Education**				
Never studied	44 (7.0)	38 (10.3)	0.32	−0.12
Fundamental	182 (28.8)	109 (29.6)		−0.018
High school	204 (32.3)	111 (30.2)		0.045
University	178 (28.2)	93 (25.3)		0.065
Graduate Studies	24 (3.8)	17 (4.6)		−0.041

Data expressed in absolute numbers and percentage in parentheses; *p*: statistical significance (chi-square test). h—Effect size Cohen’s D for continuous variables and Cohen’s h for categorical variables. A = high social class, B = high middle class. C = middle social class. D = low middle class. D = poor social class.

**Table 6:** Comparison between patients with controlled BP and uncontrolled BP in terms of clinical characteristics.

	Controlled BP			
Clinical Characteristics	Yes	No	*p*	h
	(n = 632)	(n = 368)		
**Hospital**				
Public	297 (47.0)	203 (55.2)	0.013	−0.163
Private	335 (53.0)	165 (44.8)		0.163
**Comorbidites**				
Total comorbidites	297 (47.0)	226 (61.4)	<0.001	−0.29
Diabetes mellitus	113 (17.9)	103 (28.0)	<0.001	−0.242
Dyslipidemia	183 (29.0)	157 (42.7)	<0.001	−0.287
Coronary artery disease	71 (11.2)	45 (12.2)	0.636	−0.031
Chronic kidney disease	10 (1.6)	10 (2.7)	0.216	−0.079
Stroke	23 (3.6)	23 (6.3)	0.057	−0.121
Depression	13 (2.1)	9 (2.4)	0.686	−0.026
Cardiac insufficiency	29 (4.6)	16 (4.3)	0.859	0.012
Chronic obstructive	8 (1.3)	8 (2.2)	0.27	−0.07
pulmonary disease				
Peripheral obstructive	8 (1.3)	17 (4.6)	0.001	−0.208
arterial disease				
Use of medication	256 (40.5)	208 (56.7)	<0.001	−0.322
for comorbidities				
Self-medication	221 (35.0)	192 (52.2)	<0.001	−0.349
**Purchase of the medicine**				
Health center (free)	193 (30.5)	130 (35.3)	0.065	−0.102
Popular pharmacy	135 (21.4)	89 (24.2)		−0.067
Pharmacy (Full amount)	304 (48.1)	149 (40.5)		0.153
Unscheduled visit	161 (25.5)	223 (60.6)	<0.001	−0.726
**Anxiety**				
Trait	296 (46.8)	220 (59.8)	<0.001	−0.260
State	251 (39.7)	206 (56.0)	<0.001	−0.327

Data expressed in absolute numbers and percentage in parentheses; *p*: statistical significance (chi-square test). h—Effect size Cohen’s h.

**Table 7:** Unadjusted odds ratio for factors associated with non-control of BP.

Factors Associated with not Controlling Blood Pressure			
Variable	Odds Ratio	95% CI	*p*
Age	1.09	1.000–1.021	0.059
Female	1.705	1.308–2.223	<0.001
**Social Class**			
B	0.772	0.424–1.406	0.397
C	1.12	0.642–1.952	0.69
D	1.234	0.724–2.101	0.44
E	1.16	0.695–1.935	0.57
**Comorbidities**			
Total comorbidities	1.795	1.382–2.332	<0.001
Diabetes mellitus	1.785	1.316–2.422	<0.001
Dyslipidemia	1.826	1.395–2.388	<0.001
Use of medication for comorbidities	1.921	1.481–2.493	<0.001
**Purchase of Medicine**			
Popular pharmacy	1.374	1.022–1.848	0.036
Pharmacy (full amount)	1.345	0.965–1.874	0.08
Self-medication	2.029	1.561–2.636	<0.001
**Anxiety**			
Trait	1.687	1.300–2.189	<0.001
State	1.93	1.488–2.504	<0.001
Unscheduled visit	4.499	3.417–5.924	<0.001
Self-measurement	3.705	2.827–4.856	<0.001

95% CI = 95% confidence interval. Logistic regression where the dependent variable is the lack of blood pressure control and the other independent variables; p: statistical significance (Fisher’s exact test, chi² test and Student’s *t*-test). B = high middle class. C = middle social class. D = low middle class. D = poor social class.

**Table 8:** Adjusted odds ratio and their respective 95% CI for factors associated with non-BP control.

	Adjusted Odds Ratio	95% CI	P
**Trait Anxiety**			
Female	1.544	1.161–2.053	0.003
Use of medication for comorbidities	1.749	1.324–2.311	<0.001
Self-measurement of BP	3.254	2.441–4.338	<0.001
Self-medication	1.357	1.018–1.810	0.038
State anxiety	1.587	1.200–2.098	0.001
**State Anxiety**			
Female	1.509	1.133–2.009	0.005
Use of medication for comorbidities	1.815	1.375–2.396	<0.001
Self-measurement of BP	3.352	2.515–4.468	<0.001
Self-medication	1.389	1.041–1.853	0.025
Trait anxiety	1.605	1.211–2.127	0.001

95% CI = 95% confidence interval. Logistic regression: not controlling arterial hypertension as dependent variable; independent variables: female gender, use of medication for comorbidity, self-measurement, self-medication, state/trait anxiety.

## Data Availability

The data presented in this study are available on request from the corresponding author. The data are not publicly available due to ethical restrictions.

## Appendix A. Supplementary Tables

**Table A1:** Comparison between Patients with and without Self-Measurement of Blood Pressure Regarding Sociodemographic Characteristics by Age Group.

Variables	Age Group	<45				45–54				55–64				≥65			
		Self-Measurement				Self-Measurement				Self-Measurement				Self-Measurement			
		With	Without			With	Without			With	Without			With	Without		
		(n = 47)	(n = 62)	*p*	h	(n = 76)	(n = 103)	*p*	h	(n = 134)	(n = 157)	*p*	h	(n = 190)	(n = 231)	*p*	h
**Hospital**	Public	24 (51.1)	26 (41.9)	0.438	0.183	42 (55.3)	51 (49.5)	0.454	0.115	64 (47.8)	84 (53.5)	0.348	−0.115	89 (46.8)	120 (51.9)	0.328	−0.102
	Private	23 (48.9)	36 (58.1)		−0.183	34 (44.7)	52 (50.5)		−0.115	70 (52.2)	73 (46.5)		0.115	101 (53.2)	111 (48.1)		0.102
**Gender**	Female	23 (48.9)	39 (62.9)	0.174	−0.282	49 (64.5)	50 (48.5)	0.048	0.323	81 (60.4)	81 (51.6)	0.155	0.179	116 (61.1)	132 (57.1)	0.428	0.080
	Male	24 (51.1)	23 (37.1)		0.282	27 (35.5)	53 (51.5)		−0.323	53 (39.6)	76 (48.4)		−0.179	74 (38.9)	99 (42.9)		−0.080
**Marital Status**	Married	21 (44.7)	30 (48.4)	0.961	−0.074	48 (63.2)	69 (67)	0.678	−0.080	89 (66.4)	99 (63.1)	0.802	0.070	113 (59.5)	138 (59.7)	0.010	−0.005
	Divorced	7 (14.9)	10 (16.1)		−0.034	15 (19.7)	14 (13.6)		0.166	18 (13.4)	23 (14.6)		−0.035	22 (11.6)	24 (10.4)		0.038
	Single	17 (36.2)	18 (29)		0.152	10 (13.2)	12 (11.7)		0.046	18 (13.4)	19 (12.1)		0.040	13 (6.8)	32 (13.9)		−0.233
	Widowed	0 (0)	1 (1.6)		−0.255	0 (0)	1 (1)		−0.197	5 (3.7)	11 (7)		−0.147	36 (18.9)	37 (16)		0.077
	Live with a partner	2 (4.3)	3 (4.8)		−0.028	3 (3.9)	7 (6.8)		−0.127	4 (3)	5 (3.2)		−0.012	6 (3.2)	0 (0)		0.357
**Social class**	A	0 (0)	2 (3.2)	0.031	−0.361	4 (5.3)	5 (4.9)	0.201	0.019	6 (4.5)	13 (8.3)	0.616	−0.157	19 (10)	29 (12.6)	0.111	−0.081
	B	3 (6.4)	14 (22.6)		−0.480	6 (7.9)	18 (17.5)		−0.293	20 (14.9)	18 (11.5)		0.102	18 (9.5)	34 (14.7)		−0.162
	C	14 (29.8)	15 (24.2)		0.126	18 (23.7)	17 (16.5)		0.180	21 (15.7)	26 (16.6)		−0.024	40 (21.1)	29 (12.6)		0.229
	D	17 (36.2)	11 (17.7)		0.421	26 (34.2)	26 (25.2)		0.197	36 (26.9)	37 (23.6)		0.076	41 (21.6)	49 (21.2)		0.009
	E	13 (27.7)	20 (32.3)		−0.100	22 (28.9)	37 (35.9)		−0.149	51 (38.1)	63 (40.1)		−0.042	72 (37.9)	90 (39)		−0.022
**Education**	Never studied	0 (0)	0 (0)	0.090	<0.001	2 (2.6)	3 (2.9)	0.376	−0.017	8 (6)	6 (3.8)	0.202	0.100	27 (14.2)	36 (15.6)	0.971	−0.039
	Fundamental	7 (14.9)	10 (16.1)		−0.034	13 (17.1)	26 (25.2)		−0.200	42 (31.3)	49 (31.2)		0.003	67 (35.3)	77 (33.3)		0.041
	High school	25 (53.2)	19 (30.6)		0.461	34 (44.7)	38 (36.9)		0.160	34 (25.4)	56 (35.7)		−0.224	50 (26.3)	59 (25.5)		0.018
	University	11 (23.4)	27 (43.5)		−0.431	20 (26.3)	32 (31.1)		−0.105	43 (32.1)	43 (27.4)		0.103	41 (21.6)	54 (23.4)		−0.043
	Graduate Studies	4 (8.5)	6 (9.7)		−0.041	7 (9.2)	4 (3.9)		0.220	7 (5.2)	3 (1.9)		0.184	5 (2.6)	5 (2.2)		0.031

*p*: statistical significance (chi-square test). A = high social class, B = high middle class. C = middle social class. D = low middle class. D = poor social class. h—effect size Cohen’s D for continuous variables and Cohen’s h for categorical variables.

**Table A2:** Comparison between Patients with and without Self-Measurement of Blood Pressure Regarding Clinical Characteristics.

Age Group	<45				45–54				55–64				≥65			
	Self-Measurement				Self-Measurement				Self-Measurement				Self-Measurement			
	With	Without			With	Without			With	Without			With	Without		
	(n = 47)	(n = 62)	*p*	h	(n = 76)	(n = 103)	*p*	h	(n = 134)	(n = 157)	*p*	h	(n = 190)	(n = 231)	*p*	h
Comorbidities	7 (14.9)	17 (27.4)	0.162	−0.310	39 (51.3)	45 (43.7)	0.364	0.153	83 (61.9)	72 (45.9)	0.007	0.324	127 (66.8)	133 (57.6)	0.056	0.191
Diabetes mellitus	1 (2.1)	4 (6.5)	0.388	−0.221	14 (18.4)	15 (14.6)	0.541	0.104	40 (29.9)	28 (17.8)	0.018	0.284	65 (34.2)	49 (21.2)	0.003	0.292
Dyslipidemia	5 (10.6)	12 (19.4)	0.289	−0.247	33 (43.4)	22 (21.4)	0.002	0.478	51 (38.1)	45 (28.7)	0.104	0.200	93 (48.9)	79 (34.2)	0.003	0.300
Coronary artery disease	1 (2.1)	2 (3.2)	1.000	−0.068	4 (5.3)	7 (6.8)	0.761	−0.065	18 (13.4)	14 (8.9)	0.261	0.144	40 (21.1)	30 (13)	0.035	0.216
Chronic kidney Disease	0 (0)	0 (0)			1 (1.3)	1 (1)	1.000	0.033	4 (3)	3 (1.9)	0.707	0.070	2 (1.1)	9 (3.9)	0.121	−0.192
Stroke	3 (6.4)	1 (1.6)	0.313	0.256	5 (6.6)	1 (1)	0.085	0.321	12 (9)	9 (5.7)	0.365	0.124	9 (4.7)	6 (2.6)	0.294	0.115
Depression	1 (2.1)	0 (0)	0.431	0.293	2 (2.6)	2 (1.9)	1.000	0.046	5 (3.7)	4 (2.5)	0.737	0.068	3 (1.6)	5 (2.2)	0.734	−0.043
Cardiac insufficiency	0 (0)	1 (1.6)	1.000	−0.255	2 (2.6)	9 (8.7)	0.120	−0.274	6 (4.5)	7 (4.5)	1.000	0.001	9 (4.7)	11 (4.8)	1.000	−0.001
COPD	0 (0)	0 (0)		0.000	2 (2.6)	1 (1)	0.575	0.128	4 (3)	2 (1.3)	0.419	0.121	3 (1.6)	4 (1.7)	1.000	−0.012
POAD	1 (2.1)	0 (0)	0.431	0.293	1 (1.3)	0 (0)	0.425	0.230	3 (2.2)	1 (0.6)	0.337	0.141	14 (7.4)	5 (2.2)	0.016	0.254
Use of medicationfor comorbidities	7 (14.9)	16 (25.8)	0.236	−0.273	32 (42.1)	44 (42.7)	1.000	−0.012	73 (54.5)	61 (38.9)	0.009	0.314	111 (58.4)	120 (52.2)	0.237	0.126
**Purchase of the medicine**																
Health center (free)	12 (25.5)	12 (19.4)	0.561	0.148	25 (32.9)	40 (38.8)	0.238	−0.124	44 (32.8)	44 (28)	0.586	0.105	63 (33.2)	83 (35.9)	0.484	−0.058
Popular pharmacy	9 (19.1)	9 (14.5)		0.124	18 (23.7)	14 (13.6)		0.261	35 (26.1)	40 (25.5)		0.015	50 (26.3)	49 (21.2)		0.120
Pharmacy (full amount)	26 (55.3)	41 (66.1)		−0.222	33 (43.4)	49 (47.6)		−0.083	55 (41)	73 (46.5)		−0.110	77 (40.5)	99 (42.9)		−0.047
Self-medication	22 (46.8)	11 (17.7)	0.002	0.637	33 (43.4)	27 (26.2)	0.017	0.364	84 (62.7)	53 (33.8)	<0.001	0.587	120 (63.2)	63 (27.3)	<0.001	0.738
Unscheduled visit	31 (66)	5 (8.1)	<0.001	1.320	55 (72.4)	18 (17.5)	<0.001	1.172	91 (67.9)	29 (18.5)	<0.001	1.049	127 (66.8)	28 (12.1)	<0.001	1.203
BP control	21 (44.7)	55 (88.7)	<0.001	−0.992	39 (51.3)	82 (79.6)	<0.001	−0.608	66 (49.3)	114 (72.6)	<0.001	−0.484	83 (43.7)	172 (74.5)	<0.001	−0.638
Anxiety trait	30 (63.8)	27 (43.5)	0.052	0.410	47 (61.8)	51 (49.5)	0.129	0.249	80 (59.7)	86 (54.8)	0.408	0.100	84 (44.2)	111 (48.1)	0.434	−0.077
Anxiety state	23 (48.9)	21 (33.9)	0.121	0.307	42 (55.3)	40 (38.8)	0.034	0.331	78 (58.2)	69 (43.9)	0.019	0.286	91 (47.9)	93 (40.3)	0.139	0.154

Data expressed in absolute numbers and percentage in parentheses; p: statistical significance (chi-square test). h—effect size Cohen’s h. COPD—chronic obstructive pulmonary disease; POAD–peripheral obstructive arterial disease.

**Table A3:** Comparison between Patients with Controlled BP and Uncontrolled BP in Terms of Sociodemographic Characteristics.

	Age Group	<45				45–54				55–64				≥65			
Variables		Controlled BP				Controlled BP				Controlled BP				Controlled BP			
		Yes	No			Yes	No			Yes	No			Yes	No		
		(n = 76)	(n = 33)	*p*	h	(n = 121)	(n = 58)	*p*	h	(n = 180)	(n = 111)	*p*	h	(n = 255)	(n = 166)	*p*	h
**Hospital**	Public	31 (40.8)	19 (57.6)	0.143	−0.337	58 (47.9)	35 (60.3)	0.150	−0.250	85 (47.2)	63 (56.8)	0.119	−0.191	123 (48.2)	86 (51.8)	0.487	−0.071
	Private	45 (59.2)	14 (42.4)		0.337	63 (52.1)	23 (39.7)		0.250	95 (52.8)	48 (43.2)		0.191	132 (51.8)	80 (48.2)		0.071
**Gender**	Female	42 (55.3)	20 (60.6)	0.677	−0.108	67 (55.4)	32 (55.2)	1.000	0.004	87 (48.3)	75 (67.6)	0.002	−0.392	135 (52.9)	113 (68.1)	0.002	−0.311
	Male	34 (44.7)	13 (39.4)		0.108	54 (44.6)	26 (44.8)		−0.004	93 (51.7)	36 (32.4)		0.392	120 (47.1)	53 (31.9)		0.311
**Marital Status**	Married	37 (48.7)	14 (42.4)	0.928	0.126	79 (65.3)	38 (65.5)	0.625	−0.005	120 (66.7)	68 (61.3)	0.509	0.113	150 (58.8)	101 (60.8)	0.168	−0.041
	Divorced	11 (14.5)	6 (18.2)		−0.100	19 (15.7)	10 (17.2)		−0.041	22 (12.2)	19 (17.1)		−0.139	31 (12.2)	15 (9)		0.102
	Single	24 (31.6)	11 (33.3)		−0.037	17 (14)	5 (8.6)		0.172	23 (12.8)	14 (12.6)		0.005	26 (10.2)	19 (11.4)		−0.040
	Widowed	1 (1.3)	0 (0)		0.230	1 (0.8)	0 (0)		0.182	8 (4.4)	8 (7.2)		−0.119	47 (18.4)	26 (15.7)		0.074
	Live with a partner	3 (3.9)	2 (6.1)		−0.097	5 (4.1)	5 (8.6)		−0.187	7 (3.9)	2 (1.8)		0.128	1 (0.4)	5 (3)		−0.224
**Social class**	A	2 (2.6)	0 (0)	0.612	0.326	8 (6.6)	1 (1.7)	0.329	0.257	12 (6.7)	7 (6.3)	0.599	0.015	29 (11.4)	19 (11.4)	0.411	−0.002
	B	13 (17.1)	4 (12.1)		0.142	19 (15.7)	5 (8.6)		0.219	25 (13.9)	13 (11.7)		0.065	36 (14.1)	16 (9.6)		0.139
	C	22 (28.9)	7 (21.2)		0.179	21 (17.4)	14 (24.1)		−0.168	33 (18.3)	14 (12.6)		0.159	37 (14.5)	32 (19.3)		−0.128
	D	18 (23.7)	10 (30.3)		−0.149	33 (27.3)	19 (32.8)		−0.120	45 (25)	28 (25.2)		−0.005	51 (20)	39 (23.5)		−0.085
	E	21 (27.6)	12 (36.4)		−0.188	40 (33.1)	19 (32.8)		0.006	65 (36.1)	49 (44.1)		−0.164	102 (40)	60 (36.1)		0.079
**Education**	Never studied	0 (0)	0 (0)	0.048	<0.001	3 (2.5)	2 (3.4)	0.815	−0.057	7 (3.9)	7 (6.3)	0.747	−0.111	34 (13.3)	29 (17.5)	0.217	−0.115
	Fundamental	14 (18.4)	3 (9.1)		0.275	25 (20.7)	14 (24.1)		−0.083	54 (30)	37 (33.3)		−0.072	89 (34.9)	55 (33.1)		0.037
	High school	25 (32.9)	19 (57.6)		−0.501	50 (41.3)	22 (37.9)		0.069	60 (33.3)	30 (27)		0.138	69 (27.1)	40 (24.1)		0.068
	University	31 (40.8)	7 (21.2)		0.428	34 (28.1)	18 (31)		−0.064	53 (29.4)	33 (29.7)		−0.006	60 (23.5)	35 (21.1)		0.059
	Graduate Studies	6 (7.9)	4 (12.1)		−0.142	9 (7.4)	2 (3.4)		0.179	6 (3.3)	4 (3.6)		−0.015	3 (1.2)	7 (4.2)		−0.196

Data expressed in absolute numbers and percentage in parentheses; p: statistical significance (chi-square test). h—effect size Cohen’s D for continuous variables and Cohen’s h for categorical variables. A = high social class, B = high middle class. C = middle social class. D = low middle class. D = poor social class.

**Table A4:** Comparison between Patients with Controlled BP and Uncontrolled BP in Terms of Clinical Characteristics by Age Groups.

Age Group	<45				45–54				55–64				≥65			
	Controlled BP				Controlled BP				Controlled BP				Controlled BP			
	Yes	No			Yes	No			Yes	No			Yes	No		
	(n = 76)	(n = 33)	*p*	h	(n = 121)	(n = 58)	*p*	h	(n = 180)	(n = 111)	*p*	h	(n = 255)	(n = 166)	*p*	h
Comorbidities	17 (22.4)	7 (21.2)	1.000	0.028	52 (43)	32 (55.2)	0.150	−0.245	83 (46.1)	72 (64.9)	0.002	−0.380	145 (56.9)	115 (69.3)	0.011	−0.258
Diabetes mellitus	4 (5.3)	1 (3)	1.000	0.113	18 (14.9)	11 (19)	0.519	−0.109	34 (18.9)	34 (30.6)	0.023	−0.274	57 (22.4)	57 (34.3)	0.010	−0.267
Dyslipidemia	12 (15.8)	5 (15.2)	1.000	0.018	30 (24.8)	25 (43.1)	0.016	−0.390	50 (27.8)	46 (41.4)	0.021	−0.289	91 (35.7)	81 (48.8)	0.008	−0.266
Coronary artery disease	3 (3.9)	0 (0)	0.552	0.400	7 (5.8)	4 (6.9)	0.749	−0.046	18 (10)	14 (12.6)	0.564	−0.083	43 (16.9)	27 (16.3)	0.894	0.016
Chronic kidney Disease	0 (0)	0 (0)		<0.001	1 (0.8)	1 (1.7)	0.544	−0.081	2 (1.1)	5 (4.5)	0.110	−0.217	7 (2.7)	4 (2.4)	1.000	0.021
Stroke	0 (0)	4 (12.1)	0.007	−0.711	2 (1.7)	4 (6.9)	0.088	−0.274	15 (8.3)	6 (5.4)	0.485	0.116	6 (2.4)	9 (5.4)	0.111	−0.162
Depression	0 (0)	1 (3)	0.303	−0.350	2 (1.7)	2 (3.4)	0.596	−0.116	6 (3.3)	3 (2.7)	1.000	0.037	5 (2)	3 (1.8)	1.000	0.011
Cardiac insufficiency	0 (0)	1 (3)	0.303	−0.350	8 (6.6)	3 (5.2)	1.000	0.061	7 (3.9)	6 (5.4)	0.569	−0.072	14 (5.5)	6 (3.6)	0.484	0.090
COPD	0 (0)	0 (0)		<0.001	1 (0.8)	2 (3.4)	0.246	−0.191	5 (2.8)	1 (0.9)	0.413	0.145	2 (0.8)	5 (3)	0.118	−0.172
POAD	1 (1.3)	0 (0)	1.000	0.230	0 (0)	1 (1.7)	0.324	−0.263	1 (0.6)	3 (2.7)	0.157	−0.181	6 (2.4)	13 (7.8)	0.014	−0.259
Use of medication for comorbidities	15 (19.7)	8 (24.2)	0.616	−0.109	44 (36.4)	32 (55.2)	0.023	−0.380	74 (41.1)	60 (54.1)	0.039	−0.260	123 (48.2)	108 (65.5)	0.001	−0.350
**Purchase of the medicine**																
Health Center (free)	16 (21.1)	8 (24.2)	0.090	−0.076	39 (32.2)	26 (44.8)	0.274	−0.260	48 (26.7)	40 (36)	0.175	−0.202	90 (35.3)	56 (33.7)	0.811	0.033
Popular pharmacy	9 (11.8)	9 (27.3)		−0.396	23 (19)	9 (15.5)		0.092	46 (25.6)	29 (26.1)		−0.013	57 (22.4)	42 (25.3)		−0.069
Pharmacy (Full amount)	51 (67.1)	16 (48.5)		0.379	59 (48.8)	23 (39.7)		0.184	86 (47.8)	42 (37.8)		0.201	108 (42.4)	68 (41)		0.028
Self-medication	20 (26.3)	13 (39.4)	0.182	−0.280	39 (32.2)	21 (36.2)	0.615	−0.084	71 (39.4)	66 (59.5)	0.001	−0.403	91 (35.7)	92 (55.4)	<0.001	−0.399
Unscheduled visit	13 (17.1)	23 (69.7)	<0.001	−1.123	35 (28.9)	38 (65.5)	<0.001	−0.751	51 (28.3)	69 (62.2)	<0.001	−0.694	62 (24.3)	93 (56)	<0.001	−0.660
Anxiety trait	33 (43.4)	24 (72.7)	0.006	−0.604	58 (47.9)	40 (69)	0.010	−0.430	91 (50.6)	75 (67.6)	0.005	−0.348	114 (44.7)	81 (48.8)	0.425	−0.082
Anxiety state	28 (36.8)	16 (48.5)	0.292	−0.236	44 (36.4)	38 (65.5)	<0.001	−0.592	75 (41.7)	72 (64.9)	<0.001	−0.469	104 (40.8)	80 (48.2)	0.159	−0.149

Data expressed in absolute numbers and percentage in parentheses; p: statistical significance (chi-square test). h—effect size Cohen’s h. COPD—chronic obstructive pulmonary disease; POAD—peripheral obstructive arterial disease.

**Table A5:** Association between Patients with Anxiety (Trait and State) in Terms of Clinical Characteristics Gender and Age Groups.

		Trait Anxiety				State Anxiety				
Age Group		Positive	Negative	*p*	OR (CI95%)	Positive	Negative	*p*	OR (CI95%)	Total
**<45**	**Gender**									
	**Female**	41 (71.9)	21 (40.4)	0.001	3.783 (1.699–8.422)	32 (72)	30 (46.2)	0.006	3.111 (1.366–7.088)	62 (56.9)
	**Male**	16 (28.1)	31 (59.6)			12 (27.3)	35 (53.8)			47 (43.1)
**45–54**	**Gender**									
	**Female**	57 (58.2)	42 (51.9)	0.398	1.291 (0.714–2.335)	49 (59.8)	50 (51.5)	0.271	1.396 (0.770–2.529)	99 (55.3)
	**Male**	41 (41.8)	39 (48.1)			33 (40.2)	47 (48.5)			80 (44.7)
**55–64**	**Gender**									
	**Female**	101 (60.8)	61 (48.8)	0.041	1.630 (1.020–2.606)	89 (60.5)	73 (49.3)	0.091	1.492 (0.938–2.376)	162 (55.7)
	**Male**	65 (39.2)	64 (52.1)			58 (39.5)	71 (49.3)			129 (44.3)
**≥65**	**Gender**									
	**Female**	131 (67.2)	117 (51.8)	0.001	1.907 (1.282–2.836)	120 (65.2)	128 (54.0)	0.020	1.597 (1.074–2.374)	248 (58.9)
	**Male**	64 (32.8)	109 (48.2)			64 (64.8)	109 (46.0)			173 (41.1)
	**Breslow-Day Test**				0.187				0.423	
	**Gender**									
**General**	**Female**	330 (64)	241 (49.8)	<0.001	1.834 (1.421–2.367)	290 (63.5)	281 (51.7)	<0.001	1.628 (1.262–2.099)	
	**Male**	186 (36)	243 (50.2)			167 (36.5)	262 (48.3)			

Data expressed in absolute numbers and percentage in parentheses; p: statistical significance (chi-square test or Breslow–Day test). OR—odds ratio. CI95%—95% confidence interval.
